# Central retinal vein occlusion with cerebral infarction secondary to anlotinib treatment: a case report and literature review

**DOI:** 10.3389/fphar.2023.1188218

**Published:** 2023-06-13

**Authors:** Yingying Chen, Yi Du, Lu Qiu, Jing Zheng

**Affiliations:** ^1^ Department of Ophthalmology, The Second Affiliated Hospital of Guangxi Medical University, Nanning, China; ^2^ Department of Ophthalmology, The First Affiliated Hospital of Guangxi Medical University, Nanning, China; ^3^ The Second Affiliated Hospital of Guangxi Medical University, Nanning, China

**Keywords:** anlotinib, retinal vein occlusion (RVO), cerebral infarction, adverse drug (event), case report

## Abstract

**Purpose:** We present a rare case of an elderly man with minimal pre-existing thromboses risk, who experienced central retinal vein occlusion (CRVO) and cerebral infarction after oral intake of the anti-cancer drug anlotinib, likely due to a drug-related complication.

**Observations:** A male, aged 65 years, sought care at the ophthalmology department because of acute painless 5-day vision loss in the right eye, in combination with cerebral infarction history, after oral intake of anlotinib for hepatocellular carcinoma (HCC) for over 16 months. Clinical assessment and ancillary examination verified a right eye central retinal vein occlusion diagnosis. Anlotinib is a multi-target tyrosine kinase inhibitors (TKIs) is reported to potently suppress vascular endothelial growth factor (VEGF) receptor, in order to exert strong antitumor angiogenesis and inhibit tumor occurrence. Although anlotinib is only regarded as a possible thrombosis risk factor, it is possible that anlotinib administration markedly enhanced vaso-occlusive risk within this patient.

**Conclusion and significance:** Herein, we present the first report of anlotinib-induced CRVO and cerebral infarction to our knowledge. Given our evidences, anlotinib usage is intricately linked to sight- and life-threatening thrombotic effects even among patients with reduced thrombophilic risk. Hence, patients receiving this drug must be carefully monitored for possible drug-related complications.

## Introduction

Anlotinib hydrochloride (AL3818) is a multi-target TKIs known for its strong suppression of VEGF receptor, platelet-derived growth factor receptor (PDGFR), fibroblast growth factor receptor 1-4 (FGFR1-4), c-Kit, and other kinases. In doing so, it exerts a potent antitumor angiogenesis and tumor development suppression, which greatly enhances its prospects of clinical application. Till now, several clinical trials validated the highly efficacious and safe nature of anlotinib in non-small (Han et al, 2018) and small cell lung cancer (Hu et al, 2020), soft tissue sarcoma (C. Li et al, 2021a), medullary thyroid carcinoma (D. Li et al, 2021b), as well as hepatocellular carcinoma (HCC) (He et al, 2018). However, it is also associated with various adverse events (AEs) that potentially affects the patient quality of life and hinders patient compliance to treatment. The most common anlotinib-related AEs are hypertension, fatigue, diarrhea and anorexia. However, several investigations have demonstrated particular alterations that occur in the cerebrovascular and eye.

Retinal vein occlusion (RVO) ranks second as a modulator of retinal vascular blindness after diabetic retinopathy. CRVO affects the entire retinal region; hence, it can produce far more severe vision loss than branch retinal vein occlusion (BRVO) (Scott et al, 2020). Cerebrovascular disease (CeVD) encompasses both stroke and vascular illness within the brain. It is a major contributor to global disability and mortality (Menken et al, 2000). Strong evidences suggest that a wide array of retinal vascular diseases closely associate with CeVD. Similarly, retinal venous occlusion is intricately linked to clinical CeVD (Rim et al, 2020). Drug-induced RVO and CeVD occurrences within the same patient are relatively rare. Herein, we report the first known case of CRVO and cerebral infarction secondary to anlotinib treatment.

## Case description

**FIGURE 1:**
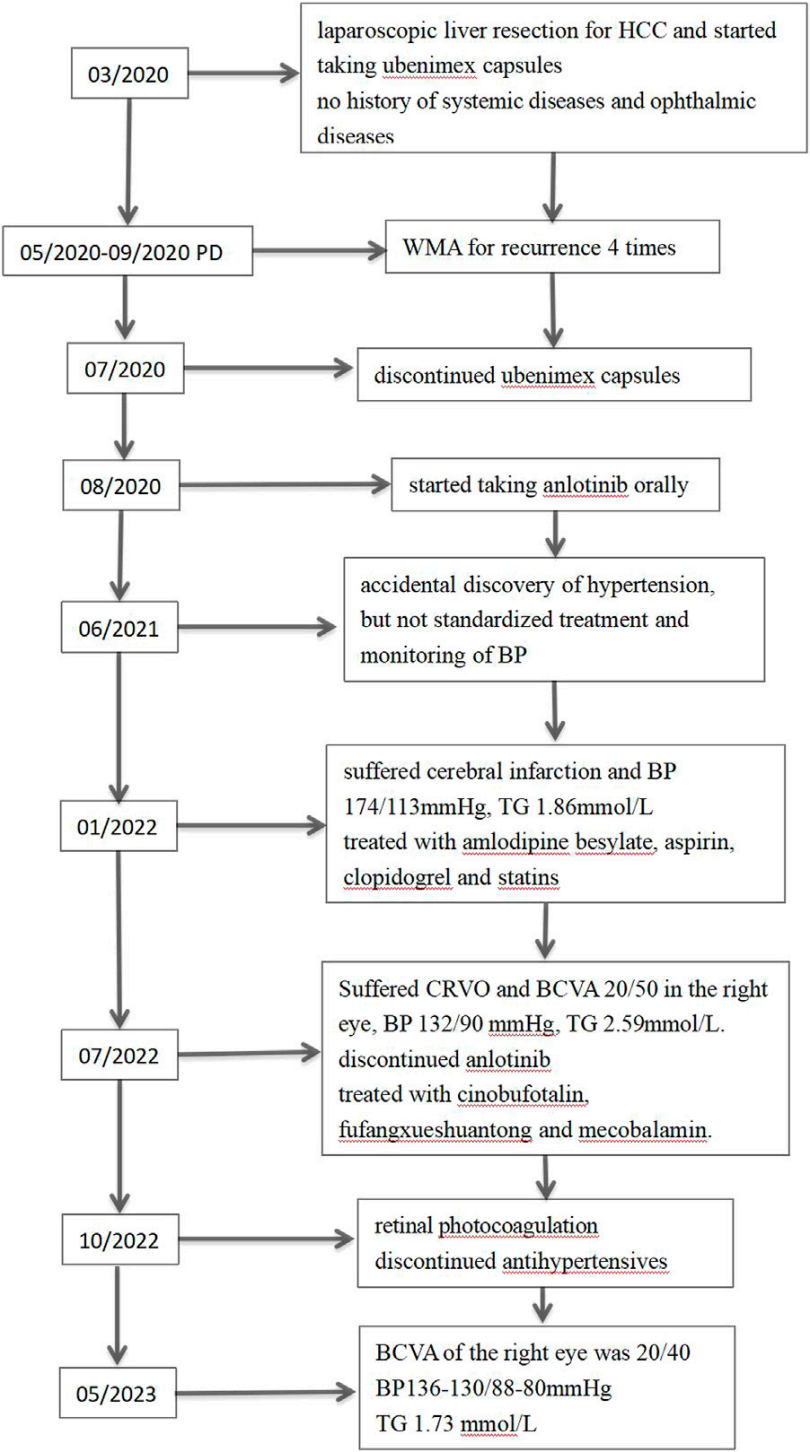
Timeline of alterations in patient condition. HCC, hepatocellular carcinoma; PD, progressive disease; WMA, microwave ablation; BP, blood pressure; TG, triglyceride; CRVO, central retinal vein occlusion; BCVA: best corrected visual acuity.

Our protocol received ethical approval from the Institutional Review Board of the Second Affiliated Hospital of Guangxi Medical University, and closely followed the guidelines recommended by the Declaration of Helsinki. We also received documented informed consent from the patient and his spouse, who permitted the disclosure of the patient identifiable information, namely, patient age, race, gender, imaging information, hematological evaluation, and medical history. Figure 1 summarizes the patient’s disease progression.

**FIGURE 2:**
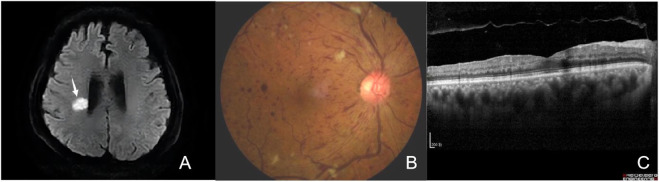
**(A)** Brain DWI. The brain DWI scan showed the brain was scattered with ischemic foc (arrows). **(B)** Fundus photography of the right eye showing tortuosity and dilatation of all branches of the central retinal vein, cotton wool patches, blot and flame-shaped hemorrhages throughout all four quadrants, without optic disc edema. **(C)** Optical Coherence Tomography (OCT) of the macula was within normal limits.

A 65-year-old nondiabetic male without a history of ophthalmic sought treatment for sudden onset painless vision diminution in the right eye for 5 days, with dark shadows in the visual field, in July 2022. Two years ago, in 2020, he was diagnosed with HCC and underwent laparoscopic hepatectomy. He had a 40-year history of hepatitis B and had been treated with entecavir. Other than that, he had no particular medical, surgical, personal or family history. Due to multiple relapses of HCC after surgery, in August 2020, he had started taking anlotinib orally 12 mg once-day, given as 2 weeks on/2 weeks off. In January 2022, he was admitted to the hospital due to “left limb weakness for 2 days”, when his blood pressure (BP) had reached 174/113 mmHg, triglyceride (TG) 1.86 mmol/L (normal value ≤ 1.70 mmol/L). Based on head magnetic resonance imaging (MRI) and diffusion weighted imaging (DWI) (Figure 2A), cerebral infarction were diagnosed. The patient was treated with amlodipine besylate (10 mg/day), aspirin (100 mg/day), clopidogrel (75 mg/day), and statins. There was no residual effect of the stroke. But his BP was still erratic. Six months later, he was readmitted due to sudden vision loss in his right eye.

At presentation the right eye best-corrected visual acuity (BCVA) was 20/50 and the left eye BCVA was 20/20. The pupils appeared equal, round, and light reactive, with no apparent defect in either eye. Binoculars motility and intraocular pressure were normal. Based on our slit-lamp evaluation, the patient had normal anterior segment. Dilated fundus assessment revealed arterial narrowing in both eyes. The right eye exhibited tortuosity and central retinal vein dilatation, cotton wool patches, blot and flame-shaped hemorrhages throughout all four quadrants, without optic disc edema, as evidenced by fundoscopy (Figure 2B).

Basic laboratory assessment revealed no aberrations in the hypercoagulability biomarkers, namely, aPTT (Activated partial thromboplastin time), PT (Pothrombin time), INR (International normalized ratio), Protein C activity, Protein-S antigen, Anti Cardiolipin IgM and IgG, B2 Glycoprotein IgM and IgG, Fibrinogen and D-dimer were assessed, and showed slight abnormality. BP was 132/90 mmHg, and TG was 2.59 mmol/L. The carotid vascular ultrasound showed plaque formation in the right subclavian vein. There was also no focal neurological, according to our neurological evaluation.

**FIGURE 3:**
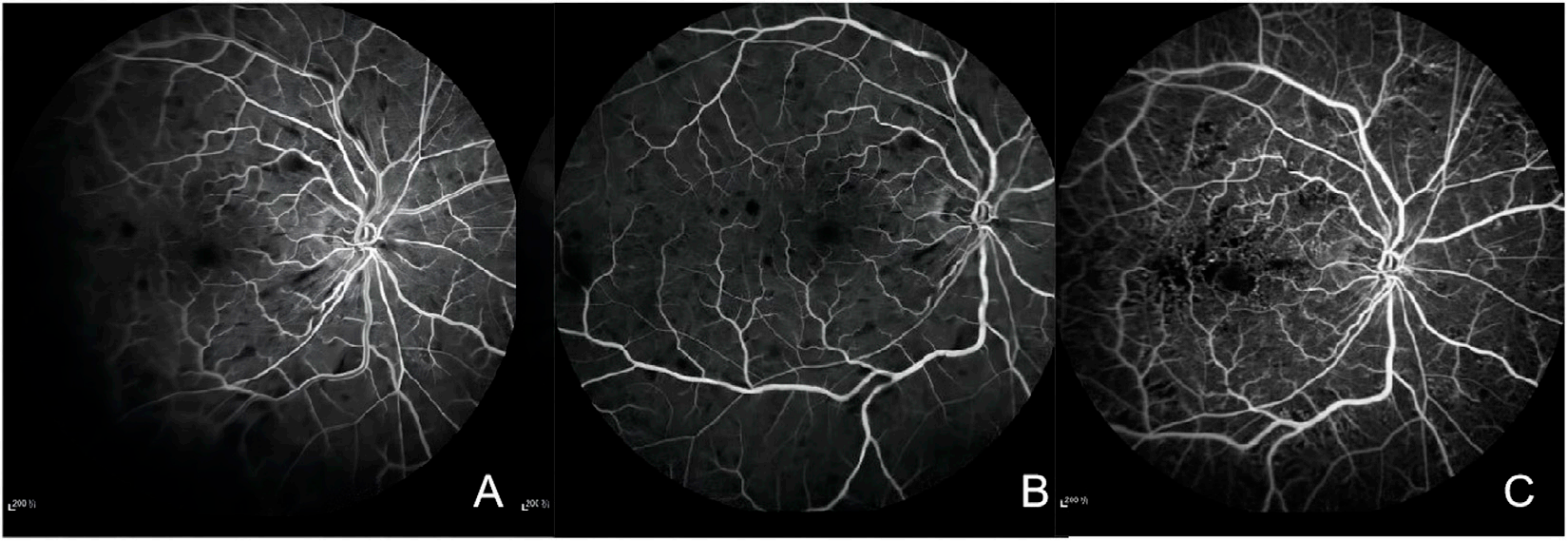
**(A)** Fluorescein angiography of the right eye in 31 s showing marked delay in venous filling, masking by retinal hemorrhages. **(B)** Fluorescein angiography of the right eye showing staining of dilated tortuous veins, stiff and thin arteries, and masking by retinal hemorrhages. **(C)** Fluorescein angiography of the right eye after 3 months showing microangioma and retinal no perfusion areas.

The macula Optical Coherence Tomography (OCT) was normal ((Figure 2C). Fundus fluorescein angiography (FFA) revealed significant arteriovenous transit delay, dilated tortuous vein staining and retinal hemorrhages-related masking (Figures 3A, B). The aforementioned manifestations agreed with a diagnosis of CRVO (Schmidt-Erfurth et al, 2019).

Based on this diagnosis and his prior medical history, anlotinib was immediately discontinued by the consulting oncologists, and replaced it with cinobufagin. Meanwhile, the patient was placed on fufangxueshuantong capsules and mocobalamin capsules to enhance circulation and facilitate nerve nourishment, as well as decrease hypertension and triglyceride, under daily BP monitoring. After 3 months, the hypertensive drugs were discontinued, and the patient BP was stable at 130–140/80–90 mmHg. Although the patient complained of lack of marked enhancement in the right eye visual acuity, but mentioned less black shadow in his vision field than before. The right eye BCVA was 20/63, and the left eye BCVA was 20//20. Based on our fundus evaluation, the retinal hemorrhage and cotton-wool spots were partially absorbed. FFA revealed there was non-perfusion zone in the retinal (Figure 3C). The foveal SD-OCT scan revealed no macular edema. Therefore, photocoagulation was performed in the retinal non-perfusion area to prevent neovascularisation and vitreous hemorrhage. Following treatment, the BCVA of the right eye was 20/40, and the fundus hemorrhage and cotton wool patches were completely absorbed at the final follow-up.

## Literature review

We screened Pubmed for rare TKIs-induced adverse outcomes during the time range of 2000-2020, and utilized the following terminology: “retinal vein occlusion and tyrosine kinase inhibitor” and “retinal vein occlusion and TKI” We found three cases of TKIs-induced RVO in four eyes in three articles. The associated clinical information are provided in Table 1.

**TABLE 1:** Review of the literature of patients who developed retinal vein occlusion following anti-cancer TKI treatment.

Author	Age/Sex	TKI drugs/dose/Tumor	Time interval between TKI treatment and onset of CRVO	Comorbidities	Management
Pyare R et al (Pyare et al, 2023)	65 y/M	axitinib 10 mg daily/renal cell carcinoma	8 months for right eye 22 months for left eye	nonhypertensive, nondiabetic history suffered a cerebrovascular accident after axitinib treatment	discontinued axitinib implanted intravitreal dexamethasone sustained release into the left eye
Schvartsman G et al (Schvartsman et al, 2016)	74 y/M	regorafenib 80 mg daily/gastrointestinal stromal tumor	11 months	hypertension, diabetes, hyperlipid emia, glaucoma before regorafenib treatment	discontinued regorafenib intravitreal injection of bevacizumab
Li ZY et al (Li et al, 2016)	42 y/M	sorafenib 800 mg daily/metastatic renal cell carcinoma	17 months	No.	discontinued sorafenib and treated with axitinib prevent inflammation, protect the optic nerve, and improve circulation

All three cases were male from India, Caucasus and China with the ages of 65, 74 and 42 year-old, respectively. All aforementioned patients were diagnosed with CRVO after an average of 14.5 months TKIs treatment (between 8 and 22 months). Two cases had no history of systemic disease, while one had a history of hypertension, diabetes, hyperlipidemia, and glaucoma prior to TKIs treatment. Two cases did not have BP description while receiving TKIs, and one case had normal BP at CRVO onset. All patients discontinued their original TKIs medication, but one switched to a different TKIs medication to continue treatment of the underlying disease. Two patients showed improvement in vision, and one had no mention of vision following TKIs treatment.

## Discussion

CRVO occurs when the central retinal vein is blocked by thrombus formation, and the general features of CRVO are retinal vein dilatation with retinal and subretinal hemorrhages, and varying degrees of retinal ischemia (Jonas et al, 2017). In a typical CRVO patient, the retina shows signs of flame-shaped or dot blot hemorrhage, as well as retinal ischemia that forms cotton-wool spots (Schmidt-Erfurth et al, 2019). CRVO diagnosis is validated based on a rise in the retinal transit duration, as evidenced FFA. The transit time of the retinal arteriovenous is usually over 5 s (Jonas et al, 2017).

Our patient had no history of vascular occlusion events and systemic diseases prior to HCC and anlotinib treatment. Additionally, the patient did not consume any other medications other than veticarn, anlotinib, and ubenmex capsulesthe for 4 months (2020.3-2020.7). There are no report that veticarn and ubenmex may cause hypertension. After 17 months of anlotinib treatment, the patient developed a sudden weakness in his left limb with elevation of blood pressure, both of which were considered adverse reactions to the drug, and brain DWI revealed acute cerebral infarction. Six months later, he suffered from blurred right eye vision, and received a CRVO diagnosis. After discontinuing anotinib and antihypertensive medications, his blood pressure remained normal, right eye vision improved, and fundus hemorrhage was completely resolved.

Predisposing factors of CRVO and cerebral infarction include hypertension, hyperlipidemia, diabetes mellitus or hypercoagulable states (Henningsen, 2001; Rehak & Wiedemann, 2010). According to a phase III clinical trial involving 437 patients (Zhou et al, 2019), hypertension occurred in 67.3% of patients treated with anlotinib, with Grade 3 or above hypertension reported in 13.6% of patients. Comparable conclusions were reached in a phase II study (Han et al, 2018). Zhang et al (Zhang et al, 2020) reported an anlotinib-induced hypertensive retinopathy in a 48- year-old woman who presented with retinal hard exudate formation and the atrophy of the outer retina, which was different from the fundus appearance in our patient. We believe that these may be the different manifestations of chronic and acute retinopathy induced by hypertension secondary to anlotinib. Hyperlipidemia is another side effect of anlotinib, which accelerates the formation of plaques and makes them unstable (Chi et al, 2018).

Anlotinib has been associated with thrombus formation in addition to the common toxicities described above (Liu et al, 2020; She et al, 2021). Microangiopathic ischemia, whether by increased peripheral vascular resistance or microthrombosis, could be involved in these rare associations and could be the mechanism underlying the findings in our case. To date, no cases of CRVO associated with anlotinib have been reported. While, several studies (Li et al, 2016; Schvartsman et al, 2016; Pyare et al, 2023) have found that other TKI drugs can induce CRVO. However, these TKIs drugs were reported to cause CRVO episodes in patients with normal BP and blood lipids except one case (Schvartsman et al, 2016) with a history of hypertension and hyperlipidemia prior to TKIs treatment. TKIs may increase the risk of thromboembolic events because TKIs potently inhibit VEGF signaling and anti-VEGF agents are known to increase the incidence of thrombosis (Scappaticci et al, 2007). VEGF plays a key role in endothelial cell proliferation and survival. Therefore, inhibition of VEGF by TKIs treatment may compromise vascular integrity (Kilickap et al, 2003) and ultimately lead to thrombosis. Meanwhile, Anti-VEGFs increase the risk of thrombosis by inducing excessive production of erythropoietin, which increases hematocrit and blood viscosity (Stahl et al, 2010). Moreover, the production of nitric oxide (NO) and prostacyclin (PGI2) could be increased by VEGF, which inhibit platelet aggregation (Zachary, 2001). Therefore, inhibition of VEGF may reduce NO and PGI-2, increasing the risk of thrombosis. Given these above-mentioned mechanisms, we speculated that the occurrence of CRVO and cerebral infarction in our patient may be the result of the combined effect of AEs and thrombosis secondary to anlotinib.

There are no current reports of treatment options for anlotinib-related CRVO. According to the dogma of ischemic retinopathy, three options are available: 1) intravitreal anti-VEGF therapy, 2) retinal photocoagulation, and 3) pars planavitrectomy for vitreous hemorrhage. There is no-perfusion region around the retina, but no edema in the macula is evidenced by FFA evaluations. We speculated that intravitreal anti-VEGF may potentially induce retinal ischemia since anti-VEGF medications may worsen retinal vascular occlusion (von Hanno et al, 2010); therefore, photocoagulation was the preferred approach. The right eye no-perfusion region coverage was managed via photocoagulation, eventually, the right eye ischemia resolved and the right eye BCVA improved.

## Conclusion

Based on our case report and literature review, without proper treatment, anlotinib can potentially damage the central nervous and ocular systems. To prevent this, early prophylactic intervention is necessary with anlotinib prescription. Furthermore, herein, we demonstrated that the early diagnosis of anlotinib-related cerebral infarction and CRVO can potentially minimize long-term neurological and visual impairment.

## Data Availability

The original contributions presented in the study are included in the article/Supplementary Material, further inquiries can be directed to the corresponding author.
